# Evaluating Toxicity and Anti-Osteogenic Activity of Artemisinin-Inspired Endoperoxides in Zebrafish Larvae

**DOI:** 10.3390/toxics14030261

**Published:** 2026-03-17

**Authors:** Yaryna S. Buzan, Gil Martins, Bruno M. S. Ferreira, Inês C. C. Costa, Maria L. S. Cristiano, Paulo J. Gavaia

**Affiliations:** 1 Centro de Ciências do Mar do Algarve (CCMAR/CIMAR-LA), Campus de Gambelas, University of Algarve, 8005-139 Faro, Portugal; a61108@ualg.pt (Y.S.B.); gsmartins@ualg.pt (G.M.); a65626@ualg.pt (B.M.S.F.); a52917@ualg.pt (I.C.C.C.); 2 Department of Chemistry and Pharmacy, Faculty of Sciences and Technology, FCT, Campus de Gambelas, University of Algarve, 8005-139 Faro, Portugal; 3 Faculty of Medicine and Biomedical Sciences, Campus de Gambelas, University of Algarve, 8005-139 Faro, Portugal

**Keywords:** artemisinin, endoperoxides, osteogenic activity, calcium homeostasis, Ca^2+^-ATPase, PfATP6, SERCA, zebrafish larvae

## Abstract

Endoperoxide-containing molecules based on the antimalarial drug artemisinin have demonstrated various biological properties, including modulation of calcium homeostasis. This study evaluated the toxicity and osteogenic activity of five newly developed tetraoxanes (YB1, YB9, YB11, YB17 and T2), alongside three of their non-peroxidic analogues (IC22, IC26 and IC33), in zebrafish (*Danio rerio*) larvae. For each compound, LC_50_ values were first determined. Behavioural responses and morphological alterations were studied as indicators of toxicological impact. The osteogenic activity was assessed through the operculum assay, followed by the analysis of gene expression markers related to calcium homeostasis (*atp2a1*), oxidative stress (*sod1*, *cat*), and osteogenesis (*sp7*, *oc2*). All the compounds evaluated induced an inhibition of osteogenic activity. T2, YB11, IC33 and IC26 affected the locomotor function by decreasing swimming activity. IC26 and IC33 induced morphological toxicity, characterized by a curved trunk and alterations in larval body curvature. From all the compounds studied, YB1, YB9, YB17 and IC22 showed selective anti-osteogenic activity, without displaying significant behavioural or morphological toxicity. In conclusion, the presence of a peroxide bond in the molecular structure of the compounds increases the anti-osteogenic activity at lower concentrations. All evaluated compounds exhibited anti-osteogenic activity and can be regarded as anti-osteogenic agents. However, YB17 did not induce transcription alterations in the genes analyzed and may thus represent the most promising compound in conditions where a controlled inhibition of bone formation is desirable.

## 1. Introduction

Artemisinin and related endoperoxide-derived compounds are of special interest due to their extensive and varied biological activities and medical applications [1,2]. Artemisinin and some of its derivatives are widely used as antimalarials, and three main hypotheses were proposed for their multitarget antiplasmodial action: the generation of toxic reactive oxygen species (ROS), which disrupt the parasite’s redox homeostasis; the generation of carbon-centred radicals, which alkylate the parasite’s proteins, and also ferriprotoporphyrin IX (heme), which interferes with its detoxification [3,4]; and the inhibition of *Plasmodium falciparum* ATPase 6 (PfATP6), a sarco/endoplasmic reticulum Ca^2+^-ATPase (SERCA) necessary for calcium homeostasis [4,5].

The major mechanism by which calcium homeostasis is disturbed and through which the parasite is killed appears to be an inhibition of PfATP6 [6]. The inhibition of Ca^2+^-ATPase can disrupt cellular functions in various ways by perturbing calcium homeostasis, leading to elevated intracellular concentrations of calcium, altered cell excitability, deranged energy metabolism, induction of apoptosis, and disruption of cellular organelles [7]. Artemisinins were found to induce similar mechanisms in other organisms, as observed in African clawed frog (*Xenopus laevis*) oocytes, where they inhibited the SERCA orthologue of PfATP6 with similar potency to thapsigargin, a known endoplasmic reticulum Ca^2+^-ATPase inhibitor [8].

Other hypotheses postulate that artemisinin disrupts the mitochondrial electron transport chain of the parasite and its SERCA. This model is based on the structural homology of artemisinin with thapsigargin and their molecular targets [9]. A multi-step process has been suggested, where artemisinin forms free radicals upon reaction with Fe^2+^ and affects PfATP6 [8]. Further, the importance of calcium homeostasis at all life stages of the parasite was emphasized through the discovery of novel putative calcium transporters in *Plasmodium falciparum* [10]. It was also shown that downregulation of plasma membrane calcium ATPase 4b (PMCA4b) in red blood cells enhances intracellular calcium and ROS, inhibiting the growth of *Plasmodium falciparum* and altering artemisinin sensitivity [11].

Artemisinin has been shown to induce the release of calcium-binding proteins from *Toxoplasma gondii*, with similar activity to the SERCA-blocker thapsigargin [12]. The disruption of calcium transport is also supported by in silico simulations, where artemisinin and its derivatives were found to bind to a distinctive pocket in PfATP6 close to the cell membrane [13]. Given the existing evidence that artemisinin inhibits Ca^2+^-ATPase in parasites, interest has grown in understanding whether artemisinin may inhibit similar Ca^2+^-ATPases in other organisms, like the hosts.

During the past decade, research has expanded into fungal cells, with the suggestion of a wider use of artemisinin and its analogues in disrupting calcium homeostasis of various organisms. Cyclic peroxides obtained from marine organisms, like plakortide F acid (PFA), have been shown to cause comparable effects in disrupting the equilibrium of calcium in fungal cells. Many hypotheses have been proposed to explain how PFA inhibits calcium ATPases and impacts vacuole-associated (Pmc1) and Golgi-associated (Pmr1) pumps, leading to toxic calcium storage inside yeast cells [14]. The molecular targets of artemisinin and its derivatives are still largely unknown, despite the efforts to determine them [12].

Previously reported effects of some artemisinin derivatives support this study and emphasize the pharmacological potential of this class of compounds [8,13,14]. We hypothesize that artemisinin analogues may delay developmental and mineralization processes by interfering with calcium transport. Given the vital role of calcium in most biological systems, including neural transmission, interference with calcium transport may lead to other physiological effects, including behavioural and locomotor changes, as well as alterations in skeletal processes.

The zebrafish (*Danio rerio*) is a widely used model in biomedical research due to its unique biological characteristics, developmental transparency, and amenity for in vivo imaging, as well as ease of genetic manipulation and availability of genomic data. Zebrafish is a recognized model for skeletal research, mostly due to the similarity in skeletal regionalization as well as in the processes of bone modelling and remodelling [15,16,17,18]. In this context, zebrafish has been used as a powerful system for studying osteogenesis and phenocopying human bone disorders, providing an efficient platform for screening anti-osteogenic compounds [19,20,21,22].

To the best of our knowledge, this study is the first to evaluate the potential of artemisinin-inspired endoperoxides as anti-osteogenic molecules. We aimed to investigate the effects of novel synthetic endoperoxides on calcium homeostasis, using zebrafish larvae as a vertebrate model, by examining whether these compounds alter Ca^2+^-ATPase activity or target other molecular pathways involved in ossification, as well as assessing their potential toxicity and biological activity—particularly their effects on osteogenic processes—in non-parasitic organisms. To evaluate the relevance of the peroxide function on activity, selected ether analogues lacking the peroxide bond were also prepared, and their properties were investigated under similar conditions.

Artemisinin and its derivatives are established antimalarial drugs. Although these drugs are comparatively safer than other antimalarial chemotypes, there may be potential risks of developmental toxicity when artemisinins are used during early pregnancy [23]. Animal and embryo models have shown reduced growth and congenital abnormalities when exposed to the artemisinin derivatives [24]. In zebrafish, these drugs may affect the production of blood cells and have been associated with morphological abnormalities during development [25,26]. This aspect will also be investigated and considered in the present study. By disclosing the first evaluation on the toxicology and biological activity of these endoperoxides in a widely used vertebrate model, our work deepens the understanding of mechanisms involved in their action and their toxicological effects, thus providing valuable information for future research.

## 2. Materials and Methods

### 2.1. Chemicals

All reagents used for synthesis were obtained from commercial sources and employed without additional purification. When required, solvents were freshly distilled using appropriate drying agents before use. Analytical thin layer chromatography (TLC) was performed on TLC Silica Gel 60 F254 aluminum plates (Merck, Darmstadt, Germany) and visualized under UV light or with suitable staining reagents, most commonly p-anisaldehyde and potassium permanganate. Column chromatography was conducted with technical grade silica gel (Sigma Aldrich, Darmstadt, Germany; pore size 60 Å, mesh particle size 230–400, 40–63 µm particle size).

### 2.2. Analytical Methods for Structural Characterization of Synthesized Compounds

^1^H and ^13^C Nuclear Magnetic Resonance (NMR) spectra were recorded on a JEOL 500 MHz system (JEOL Ltd., Tokyo, Japan) equipped with a Royal HFX triple resonance probe, using the indicated deuterated solvents. Chemical shifts (δ) are reported in parts per million (ppm) relative to tetramethylsilane (TMS) as the internal standard. Melting points (°C) were measured using an SMP30 apparatus (Bibby Scientific, Stone, UK) and are uncorrected. Mass spectrometry analyses were performed on an Orbitrap Elite (Thermo Scientific, Bremen, Germany) mass spectrometer, featuring a linear ion trap (MS^n^, *n* = 2–10) and a high-field orbitrap (up to 240,000 resolution), which can operate independently or in combination. Higher-energy collisional dissociation (HCD) was also available. The mass spectrometer was operated using a Heated ESI (HESI-II) ion source with the following parameters: heater temperature 50 °C, sheath gas flow 10 arbitrary units, auxiliary gas flow 2 arbitrary units, capillary temperature 325 °C, spray voltage 3.2 kV, S-Lens RF level 60%, and a scan range of 50–1500 *m*/*z*. Substrate solutions were infused at a flow rate of 5 μL/min.

### 2.3. Synthesis

The 1,2,4,5-tetraoxanes and their ether analogues (Table 1) were synthesized by adapting procedures previously described in the literature. The detailed procedures and the experimental conditions for synthesis and chemical characterization of the compounds are detailed in the Supplementary Materials S1 and S2.

### 2.4. Zebrafish Larvae Maintenance

A breeding stock of wild-type (WT) zebrafish (AB strain; ZFIN ID: ZDB-GENO-960809-7) maintained at the Centre of Marine Sciences (CCMAR, Faro, Portugal) for over ten generations served as the source of embryos for this experiment. The zebrafish facility was operated under a controlled 14 h light/10 h dark photoperiod, ambient humidity of approximately 60%, and a room temperature of 24 ± 1 °C. The holding system consisted of 3.5 L tanks connected to a 980 L recirculating housing system (ZebTEC; Tecniplast, Buguggiate, Italy).

Water quality was maintained through biological filtration (ceramic beads), mechanical filtration (pleated cartridge filters, 50 µm), activated carbon filtration, and ultraviolet sterilization (180,000 µWs/cm^2^). Approximately 10% of the total water volume was renewed daily. System water was kept at 28 ± 1 °C, with a pH of 7.5 ± 0.2 and conductivity of 750 ± 30 µS/cm. Nitrite (NO_2_^−^) and ammonium (NH_4_^+^) concentrations were monitored weekly and maintained below 0.1 mg/L, while nitrate (NO_3_^−^) levels remained under 50 mg/L throughout the experimental period, as described by Martins et al. (2019, 2020) [27,28]. Adult fish were fed twice daily (9 a.m. and 3 p.m.) with a microdiet corresponding to 7–15% of fish body weight (Zebrafeed, Sparos Lda, Olhão, Portugal) whose nutritional profile is described in Castro et al. (2025) [29].

Adult fish were mated to obtain larvae. After spawning, embryos were collected and maintained at a density of 100–200 eggs per 1 L tank in E2 medium. Embryos were incubated in a SANYO MIR-153 incubator at 28 °C until use in experimental procedures.

### 2.5. Toxicity Testing and LC_50_ Calculation

To evaluate the toxicity of the synthesized endoperoxides, we have determined the concentration that causes 50% lethality (LC_50_) through concentration–response assays in zebrafish larvae (Figure S1). Initially, four concentrations (100, 50, 25, and 5 μM) were assayed for each compound. Follow-up assays were performed with optimized, narrower dose response ranges, which were tested based on the mortalities recorded in the first assay, to accurately determine the LC_50_ value for each compound.

For each compound concentration assessed, WT-AB larvae 3 days post-fertilization (dpf) were distributed into triplicates on a 12-well plate. Zebrafish larvae (*n* = 9) were placed in each well containing 2 mL of a treatment solution composed of fish water with the corresponding compound concentration in solution. The exposure was conducted for 72 h. During this period, 70% of the treatment solution was changed daily to ensure adequate water quality and stability of the compounds.

Mortality was recorded at 24, 48, and 72 h of exposure. Based on the cumulative mortality values obtained at 72 h, the LC50 values for every compound were calculated using AAT Bioquest LC50 calculator (AAT Bioquest, Inc. 2024. Quest Graph™ LC50 Calculator (AAT Bioquest, Inc., Pleasanton, CA, USA). Available at: “https://www.aatbio.com/tools/ld50-calculator (accessed on 5 November 2024)”.

### 2.6. Assessment of Mineralization Using the Operculum System

Following the determination of LC_50_ values, mineralization assays were performed on zebrafish larvae using compound-specific concentration ranges below the LC_50_.

Larvae of WT-AB zebrafish at 3 dpf were transferred to 12 well-plates at a density of 5 larvae in 5 mL of water. The larvae were exposed to a range of concentrations of each compound: YB1 (1, 6, 30 µM), YB9 (0.5, 2, 8 µM), YB11 (1, 6, 30 µM), YB17 (0.5, 2, 6 µM), IC22 (1, 4, 20, 30 µM), IC26 (12, 36, 60 µM), IC33 (2, 8, 40 µM), T2 (0.5, 2, 5 µM). Ethanol (Merck, Darmstadt, Germany) (0.1%) and dimethyl sulfoxide (DMSO; Sigma-Aldrich, St. Louis, MO, USA) (0.1%) were used as negative controls (solvents), and calcitriol at 10 pg/mL (Sigma-Aldrich, St. Louis, MO, USA) was used as a positive control. The treatment medium was renewed (70% of the total volume) daily until the end of the treatment period.

Zebrafish larvae were anesthetized with 0.186 mg/mL of MS-222 (tricaine methanesulfonate, 0.6 mM, pH 7.0, Sigma-Aldrich, St. Louis, MO, USA) to ensure immobilization during imaging. Larvae were stained for 15 min, at room temperature, with 0.01% alizarin red S (AR-S) prepared in Milli-Q water (pH 7.4) and washed twice with Milli-Q water for 5 min, following the methodology described in Tarasco et al., 2017 [30].

High-resolution images were acquired using an MZ10F fluorescence stereomicroscope (Leica, Wetzlar, Germany) coupled to a DFC7000T colour camera (Leica), equipped with an integrated high-intensity fluorescence illumination system. Fluorescence images were captured using the ET560/40x–ET630/75m filter set (Leica, Wetzlar, Germany), with an exposure time of 600 ms. Images were acquired using the following parameters: optical zoom set to 5.0×, gain 3.5, and exposure time 1086 ms.

Lateral images of the head region were analyzed using the ZFBONE toolset for Fiji/ImageJ software (v.154k) following the methodology described in Tarasco et al., 2020 [31]. Operculum area was manually outlined with the assistance of the “Operculum assay” tool and the polygon selection tool, using landmarks under fluorescence contrast. Normalized area of operculum was calculated by determining the ratio of area of operculum to the area of head.

### 2.7. Morphological Analysis

Morphometric analysis was evaluated to identify possible treatment-induced effects on growth, assessed by total body length (Figure S2A), and on the angle of body curvature (Figure S2B) of larvae.

Larvae at 6 dpf, treated with the compounds represented in Table 1, at the lowest concentration of each compound that exhibited osteogenic activity, and with the negative controls (DMSO and ethanol), were anesthetized, as described above, and oriented laterally for imaging. Three biological replicates of 5 larvae were used per treatment group.

Images were acquired as previously described, under standardized imaging conditions: optical zoom 2.0×, gain 2.4, and exposure time 192.41 ms. Larval total length was measured from the tip of the snout to the posterior end of the caudal fin using the “Straight” tool in Fiji/ImageJ by tracing the body with consecutive straight segments and summing their lengths.

Curvature angle was measured using the “Angle” tool in Fiji/ImageJ, tracing three points along the body axis, one at the anterior end of the head, one at the point of maximum curvature along the notochord, and one at the posterior end of the tail. The internal angle was measured as the curvature angle.

Larvae were imaged in dorsal view, ensuring correct eye-to-eye and parallel alignment to the camera plane to ensure reproducible angular measurement.

### 2.8. Behaviour Analysis

Zebrafish larvae at 6 dpf were used to determine alterations of behaviour after experimental exposure to the compounds. Eight treatment groups (YB1, YB9, YB11, YB17, T2, IC22, IC26 and IC33) and two negative control groups (DMSO and Ethanol) were tested at the lowest concentration of each compound that exhibited osteogenic activity. Each group was run in triplicate with 15 larvae per replicate. Single larvae were added to each well of a standard 24-well plate, containing 2 mL of system water. The plate was allowed a 10 min acclimation period to reduce handling stress and hyperactivity. After acclimation, the plate was loaded into the Zantiks MWP automated testing unit (Zantiks Ltd., Cambridge, UK), where locomotor behaviour was tracked for 5 min under constant illumination (i.e., 100 lux white light) following the methodology described in Varela et al. (2022) [32].

The Zantiks tracking software recorded x-y coordinates of each larva every second during the 5 min trial. Monitoring was conducted at a standard frame rate (25 fps) and spatial resolution adequate for effective detection of larval movement within wells. Raw tracking data were stored as CSV files containing time-stamped coordinate values per individual. Locomotor behaviour was quantified as total distance moved (pixels) per larva, as the cumulative distances between successive coordinates. Pixel distance was used as a locomotor activity surrogate, as commonly used in larval zebrafish assays when absolute spatial calibration is not required.

### 2.9. Expression of Marker Genes

Three replicates of 15 larvae at 6dpf were collected from each experimental condition, and total RNA was extracted using NZYol (NZYTech, Lisbon, Portugal) following the manufacturer’s protocol. RNA purity and concentration were analyzed on a NanoDrop OneC spectrophotometer (ThermoFisher Scientific, Madrid, Spain). One microgram of total RNA was treated with RQ1 RNase-free DNase and subsequently reverse transcribed using MMLV-RT (Invitrogen, Thermo Fisher Scientific, Waltham, MA, USA) using an oligo(dT)-adapter primer. Real-time quantitative PCR (qPCR) was then performed with 10 ng of cDNA, gene-specific primers (see Supplementary Table S1), and the SensiFAST™ Probe No-ROX Kit (Bioline Meridian Bioscience, Meridian Bioscience Inc., Memphis, TN, USA), following the manufacturer’s recommendations. Amplification reactions were performed on a CFX96 thermocycler (Bio-Rad Laboratories, Hercules, CA, USA) under the following cycling conditions: an initial denaturation at 95 °C for 2 min, followed by 37 cycles of 95 °C for 10 s and 65 °C for 20 s. Primer efficiencies exceeded 95%, and specificity was confirmed by sequence analysis and melting curve inspection after each qPCR run. Relative transcript abundance was calculated using the 2-ΔΔCt method following the methodology described in Livak et al., 2001 [33].

To select reference genes with stable expression for normalization in qPCR analysis, expression levels of three widely used housekeeping genes—18S ribosomal RNA (*18s*), beta-actin (*β-actin*), and elongation factor 1-alpha (*ef1α*)—were tested in representative cDNA samples. *18S* ribosomal RNA showed the most stable expression for these experimental conditions and was therefore selected as the housekeeping gene for normalization of qPCR gene expression analysis.

For gene expression analysis by qPCR, cDNA from larvae exposed to three test compounds—IC22, YB1, and YB17—at the lowest concentration of each compound that exhibited osteogenic activity, and a negative control (DMSO) was used. The expression levels of marker genes for oxidative stress response (i.e., *catalase*, *cat*; *superoxide dismutase 1*, *sod1*) and for bone and skeletal muscle development (i.e., *osterix*, *sp7*; *osteocalcin 2*, *oc2*; *sarco*/*endoplasmic reticulum Ca^2+^-ATPase 1*, *atp2a1*; *alkaline phosphatase*, *biomineralization associated*, *alpl*; *collagen*, *type I alpha 1a*, *col1a1a*; *and runt-related transcription factor 2b*, *runx2b*) were quantified. All qPCR assays were repeated three times using biological replicates (*n* = 3).

### 2.10. Statistical Analysis

**Figure 1:**
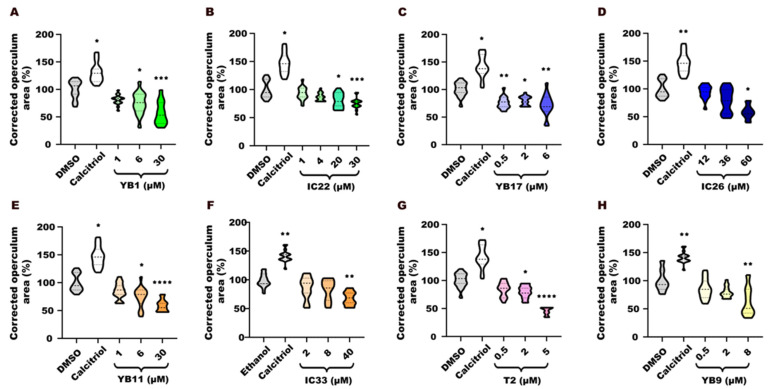
(**A**–**H**) Anti-osteogenic effects on the operculum of 6 days post-fertilization (dpf) zebrafish larvae exposed to 1,2,4,5-tetraoxanes (YB1, YB9, YB11, YB17, and T2) and some corresponding control molecules lacking the peroxide bond (IC22, IC26, and IC33). The effects of the compounds were evaluated through analysis of the corrected operculum area (ratio of operculum area/head area) and expressed as percentage relative to the negative control (DMSO, or ethanol for IC33). Data normality was assessed using the Anderson–Darling test (*p* < 0.05). Each treatment group was compared with its respective vehicle control, using one-way ANOVA followed by Kruskal–Wallis test. Statistical significance is indicated using asterisks: *p* < 0.05 (*), *p* < 0.01 (**), *p* < 0.001 (***), and *p* < 0.0001 (****).

**Figure 2:**
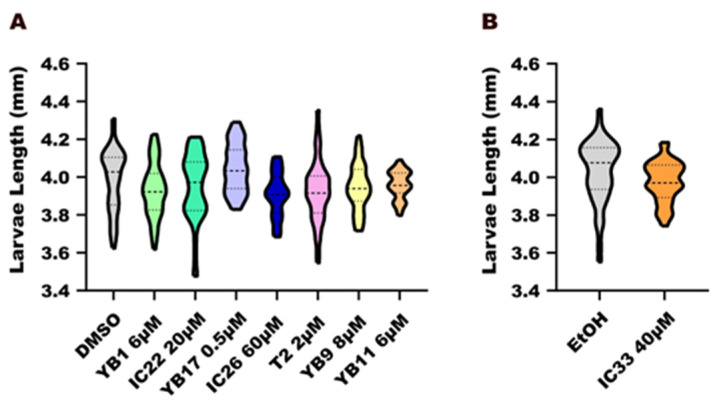
Morphological assay of zebrafish larvae at 6 days post-fertilization (dpf). Larval total length (**A**,**B**) after exposure to endoperoxide compounds (YB1, YB9, YB11, YB17, T2) and their control molecules without the peroxide bond (IC22, IC26, IC33), compared with negative controls (DMSO or ethanol—EtOH). Data normality was assessed using the Anderson–Darling test (*p* < 0.05). Each treatment group was compared with its respective vehicle control, using either one-way ANOVA followed by the Kruskal–Wallis test (**A**) or by unpaired *t*-test (**B**).

**Figure 3:**
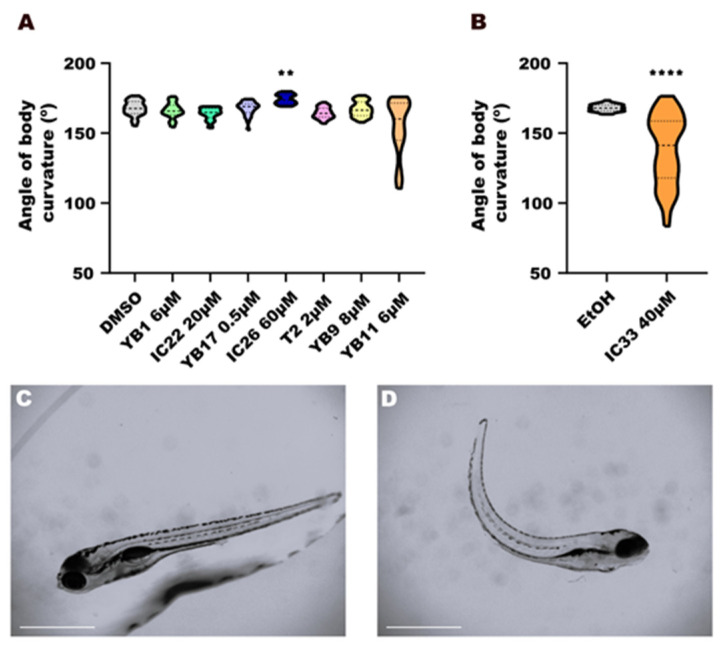
(**A**,**B**) Morphological assay of zebrafish larvae at 6 days post-fertilization (dpf). Larval body curvature after exposure to endoperoxide compounds (YB1, YB9, YB11, YB17, T2) and their control molecules without the peroxide bond (IC22, IC26, IC33), compared with negative controls (DMSO or ethanol—EtOH). Data normality was assessed using the Anderson–Darling test (*p* < 0.05). Each treatment group was compared with its respective vehicle control, using either one-way ANOVA followed by the Kruskal–Wallis test (**A**) or by unpaired *t*-test (**B**). Statistical significance is indicated using asterisks: *p* < 0.01 (**),and *p* < 0.0001 (****). (**C**,**D**) Lateral view of typical morphological characteristics of zebrafish larvae (DMSO) (C) and lateral view of abnormal morphological characteristics of zebrafish larvae (as example, IC33 40 µM) (**D**). Scale bar: 1 mm.

**Figure 4:**
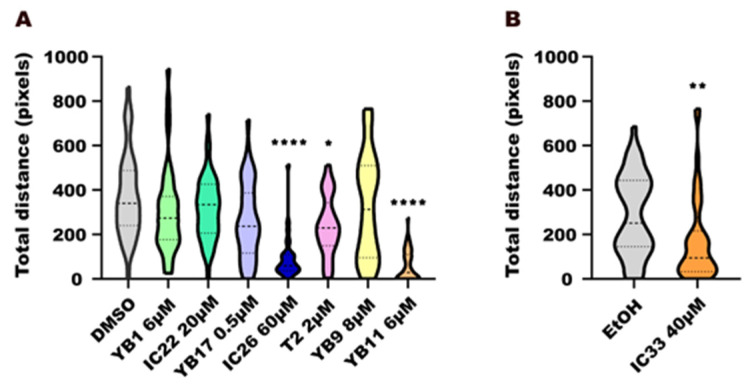
(**A**,**B**) Zebrafish larval behaviour test. Total distance travelled by larvae upon exposure to endoperoxide compounds (YB1, YB9, YB11, YB17, T2) and their peroxide bond-lacking control molecules (IC22, IC26, IC33), compared to negative controls (DMSO or ethanol). Data normality was assessed using the Anderson–Darling test (*p* < 0.05). Each treatment group was compared with its respective vehicle control, using either one-way ANOVA followed by Kruskal–Wallis test (**A**) or by unpaired *t*-test (**B**). Statistical significance is indicated using asterisks: *p* < 0.05 (*), *p* < 0.01 (**), and *p* < 0.0001 (****).

**Figure 5:**
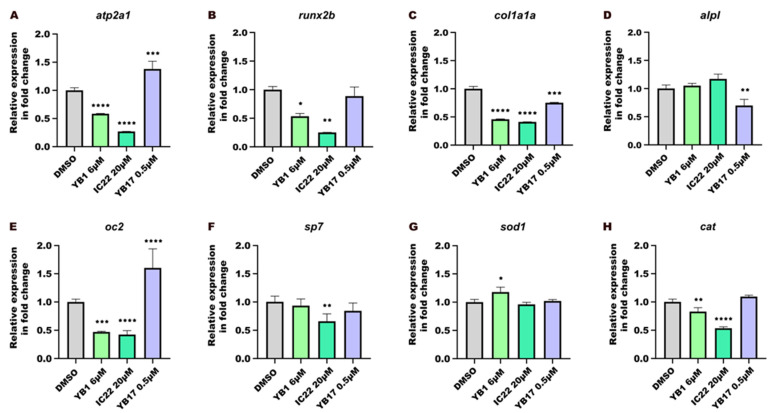
Relative expression levels of genes *atp2a1* (**A**), *runx2b* (**B**), *col1a1a* (**C**), *alpl* (**D**), *oc2* (**E**), *sp7* (**F**), *sod1* (**G**), and *cat* (**H**) in 6 days post-fertilization (dpf) zebrafish larvae following treatment with tetraoxane compounds and one control analogue. Levels of gene expression were quantified by qPCR in YB1-treated (6 μM), IC22-treated (20 μM; peroxide bond-deficient analogue of YB1), and YB17-treated (0.5 μM) zebrafish larvae at 6 dpf. Data is presented as mean ± SD. Data normality was assessed using the Anderson–Darling test (*p* < 0.05). Each treatment group was compared with DMSO (negative control), using one-way ANOVA followed by a post hoc test (Dunnett’s multiple comparison test). Statistical significance is indicated using asterisks: *p* < 0.05 (*), *p* < 0.01 (**), *p* < 0.001 (***), and *p* < 0.0001 (****).

Statistical analyses were carried out using Prism software (version 6.00; GraphPad Software, La Jolla, CA, USA), and the data are presented as mean ± SD. Data normality was evaluated using the Anderson–Darling test (*p* < 0.05) for all datasets used in the graphical analyses. Differences among groups were evaluated by one-way ANOVA followed by Kruskal–Wallis test multiple comparison test (Figure 1, Figure 2A, Figure 3A and Figure 4A) or by unpaired *t*-tests (Figure 2B, Figure 3B and Figure 4B) or by a post hoc test (Dunnett’s multiple comparison test) (Figure 5). Statistical significance is indicated using standard asterisk notation: *p* < 0.05 (*), *p* < 0.01 (**), *p* < 0.001 (***), and *p* < 0.0001 (****).

## 3. Results

### 3.1. Toxicity of Endoperoxides and LC_50_

The results indicated that the lethal effective concentrations for 50% of the population (LC_50_) varied quite considerably in the different artemisinin-derived endoperoxides tested (Table 2), with YB2 and T2 presenting higher toxicity, as shown by the lowest LC_50_ values of 5 and 5.2 µM, respectively, and IC26 presenting the highest LC_50_ value of 74.4 µM (Table 2).

Although all the concentrations assessed in this assay were below the respective LC_50_ values, mortality was still observed at the highest test concentrations, even though the mortality rates did not reach 50%.

### 3.2. Assessment of Mineralization

To determine the osteogenic effects of the synthesized endoperoxide compounds, we performed an operculum assay on zebrafish larvae, exposed from 3 to 6 dpf following the methodology described in Tarasco et al., 2017 [30]. The operculum area was normalized considering each larva head area, and the results were compared across different drug treatments (Figure S3A,B), with DMSO or ethanol as negative controls (Figure S3C,D) and calcitriol as a positive control (Figure S3E,F).

As expected, calcitriol treatment induced an increase in operculum area compared to negative controls in all experiments (Figure 1A–H). YB1 treatment induced a dose-dependent effect on operculum formation (Figure 1A). At 6 and 30 µM YB1, the operculum area of larvae was significantly reduced compared to the negative control (*p* < 0.05 and *p* < 0.001, respectively), which is consistent with an anti-osteogenic effect. The corresponding non-peroxidic control IC22 (Figure 1B) showed no significant effects over the operculum area relative to negative controls at lower concentrations (1 and 4 µM), but higher concentrations (20 and 30 µM) induced a significant reduction in the mineralized operculum (*p* < 0.05 and *p* < 0.001, respectively).

YB17, assayed as a salt, also showed inhibitory activity on the operculum (Figure 1C). A statistically significant reduction in operculum mineralized area was observed for all concentrations tested (0.5, 2 and 6 µM) (Figure 1C). The neutral ether control IC26 (Figure 1D) only induced an anti-osteogenic effect (*p* < 0.05) at the higher concentration tested (60 µM).

YB11 induced a significant inhibition of operculum growth at 6 and 30 µM, relative to negative controls (*p* < 0.05 and *p* < 0.0001) (Figure 1E).

IC33 (Figure 1F) inhibited mineralization (*p* < 0.01) only at the higher concentration (40 µM). T2 (Figure 1G) induced a statistically significant decrease in the operculum area in larvae treated at 2 µM (*p* < 0.05) and 5 µM (*p* < 0.0001) compared to negative controls. YB9 (Figure 1H) induced a statistically significant decrease in the operculum area in larvae treated at 8 µM (*p* < 0.01) compared to negative controls, but not at lower concentrations.

### 3.3. Morphological Evaluation

#### 3.3.1. Larval Length

In the control groups, larvae presented an average total length 4.0–4.2 mm. Zebrafish larvae treated with all tested compounds showed no differences in growth compared to the control groups (Figure 2A,B).

#### 3.3.2. Body Curvature

The larvae from control groups presented values for angles of body curvature close to 170°, reflecting a normal body axis alignment. In contrast, IC26 (60 µM) induced a phenotype that was characterized by excessive body curvature (*p* < 0.01). Affected larvae showed an increased angle in the trunk (Figure 3A), characteristic of developmental toxicity or neuromuscular dysfunction. IC33 (40 µM) also caused increased body curvature (Figure 3B), greater than the control (DMSO; *p* < 0.0001), and the deformation was more severe than that observed for IC26. The other endoperoxides (YB9, YB17, T2) did not cause significant alterations of body shape, indicating preservation of morphology at the tested concentrations.

After the larvae were exposed to all the compounds, malformations were evident particularly in larvae from the groups treated with IC33 (40 µM) and IC26 (60 µM) were compared to DMSO-treated control larvae (Figure 3C). These malformations were assessed by measuring deviations of the angle of body axis, where we observed a marked curvature of the larvae in the IC33 group (Figure 3D) and a moderate curvature identified in larvae from group IC26, as shown in Figure 3A.

### 3.4. Behaviour Essay

In order to more closely investigate one of the potential systemic or neurotoxic side effects that could be associated with the altered body curvature observed after exposure to the tested compounds, we evaluated larval motor activity at 6 dpf, through the measuring of distance travelled during the course of the behavioural assay. All the groups were normalized to their respective negative controls. The concentrations used in this assay were the lowest concentration where an anti-osteogenic effect was observed in the operculum assay.

As expected, the negative controls provided strong locomotor responses with mean distances of 250–350 pixels, and these were used as the standard for comparisons. Regarding the synthetic compounds under investigation, exposure to ether IC26 at 60 µM produced inhibition of locomotor activity (*p* < 0.0001), as larvae swam significantly shorter distances than the larvae treated with DMSO (Figure 4A). Notably, exposure to tetraoxane YB11 at 6 µM caused inhibition of movement (*p* < 0.0001), with larvae showing nearly complete abolition of swimming activity. Exposure to tetraoxane T2 at 2 µM (*p* < 0.05) and ether IC33 at 40 µM (*p* < 0.01) also showed a statistically significant reduction in activity, although to a lesser extent (Figure 4A,B).

Exposure to YB1 at 6 µM, IC22 at 20 µM, YB17 at 0.5 µM, and YB9 at 8 µM did not affect the locomotion, compared to the negative control (DMSO).

### 3.5. Gene Expression Analysis

The relative expression levels of selected marker genes *atp2a1* (Figure 5A), *runx2b* (Figure 5B), *col1a1a* (Figure 5C), *alpl* (Figure 5D), *oc2* (Figure 5E), *sp7* (Figure 5F), *sod1* (Figure 5G), and *cat* (Figure 5H) were evaluated in 6 dpf zebrafish larvae, following treatment with tetraoxanes YB1 (6 μM) and YB17 (0.5 μM) and one control analogue, the ether IC22 (20 μM).

Treatments with the compounds that showed only an osteogenic effect were selected for gene expression analysis and were shown to induce considerable alterations on the expression levels of marker genes involved in musculoskeletal development and oxidative stress response in the exposed zebrafish larvae. Treatment with YB1 resulted in a significant downregulation of *atp2a1* (*p* < 0.0001, Figure 5A), which is involved in calcium transport; *runx2b* (*p* < 0.05, Figure 5B), a key regulator of osteoblast differentiation; *col1a1a* (*p* < 0.0001, Figure 5C), a major structural gene involved in collagen matrix formation in zebrafish larvae; *oc2* (*p* < 0.001, Figure 5E), a marker of mature osteoblasts; and *cat*, a gene associated with the antioxidant defence system (*p* < 0.01, Figure 5H). The expression of *alpl* (Figure 5D), a gene involved in osteoblast maturation and mineralization and the immature osteoblast marker *sp7* (Figure 5F), remained unaltered and the treatment with YB1 resulted in upregulation of *sod1*, another gene associated with the antioxidant defence system (*p* < 0.05, Figure 5G). IC22, the non-peroxidic analogue of YB1, caused a strong downregulation of *atp2a1* (*p* < 0.0001, Figure 5A), *runx2b* (*p* < 0.01, Figure 5B), *col1a1a* (*p* < 0.0001, Figure 5C), *oc2* (*p* < 0.0001, Figure 5E), *sp7* (*p* < 0.01, Figure 5F) and *cat* (*p* < 0.0001, Figure 5H), but did not affect *alpl* (Figure 5D) and *sod1* (Figure 5G) expression. YB17, the salt version of the endoperoxide scaffold, induced a downregulation of *col1a1a* (*p* < 0.001, Figure 5C) and *alpl* (*p* < 0.01, Figure 5D) and an upregulation of *atp2a1* (*p* < 0.001, Figure 5A) and *oc2* (*p* < 0.0001, Figure 5E).

### 3.6. Resume

At the phenotypic level, YB1, YB17, and IC22 induced an effect in the osteogenic assay, but with no observable changes in behaviour, larval length, and body curvature. This was in sharp contrast to other molecules screened (e.g., IC26, YB11, IC33, T2, YB9), where a variety of toxic effects were observed, such as impaired locomotor activity, growth reduction in larvae, and increased morphological abnormalities. The selective anti-osteogenic effects of YB1, YB17, and IC22, without induction of systemic developmental toxicity, make these three molecules the most promising candidates for future studies (Table 3).

## 4. Discussion

Taken together, our findings indicate that the artemisinin-derived 1,2,4,5-tetraoxanes, as well as several peroxide-free control molecules, exhibit anti-osteogenic activity and toxicity in zebrafish larvae, providing evidence that these compounds are teratogenic. Teratogenic effects have been reported for other artemisinin-derived molecules [23,24,25,34]. According to Clark 2009 [24], artemisinin derivatives including dihydroartemisinin (DHA), artemether, arteether and artesunate have been found to induce embryonic lethality in several animal models, including rodents and primates. Exposure to DHA (1–10 mg/L) caused dose-dependent abnormal phenotypes in the early developmental stages of zebrafish [35]. A central role is played by the cleavage of the endoperoxide bridge, which produces ROS and initiates oxidative damage. In vitro studies have demonstrated that DHA triggers the apoptotic cascade by disrupting calcium homeostasis and inducing oxidative stress, in addition to affecting porcine oocyte maturation by altering cytoskeletal dynamics [34].

It is well documented that artemisinin derivatives interfere with calcium homeostasis [36,37]; however, to the best of our knowledge, this is the first reported study evaluating the effects of calcium imbalance by skeletal assessment, using the established operculum assay, and expression analysis of genes involved in calcium regulation.

In order to evaluate skeletal effects, we used zebrafish larvae as the animal model. Zebrafish larvae have previously been used as models to study teratogenic effects caused by artemisinin derivatives, where it was shown that it induces erythrocyte loss through apoptotic pathways during early development [25,26].

All endoperoxides tested (YB1, YB9, YB11, YB17, and T2) and the non-peroxidic control compounds (IC22, IC26, IC33) evidenced clear anti-osteogenic activity, although with variable dose–response profiles. Non-peroxidic control compounds, IC22 and IC33, showed inhibition at higher doses than the corresponding peroxidic ones, YB1 and YB11. This indicates that the presence of a peroxide bond in the structure of the molecule is most likely related to the inhibitory activity over osteogenesis, as observed in the formation of the mineralized operculum in zebrafish larvae.

On the other hand, the analogue IC26 exhibited higher anti-osteogenic activity than the parent salt YB17 at the tested concentrations. Nevertheless, this discrepancy could be due to the concentrations of the compounds, which differed by a factor of 120.

Interestingly, both the amide-functionalized endoperoxides, T2 and YB9, exhibited high anti-osteogenic activity at relatively low concentrations, suggesting that the amide functionality could be an important determinant of activity and toxicity.

Prominent deformities—particularly a curved trunk—were observed after treatment with control molecules lacking the peroxide bond, such as IC26 and IC33. According to the literature, several studies have described morphological alterations as result of teratogenic effects following treatment with artemisinin and its derivatives. After the administration of a single oral dose of artesunate, DHA, arteether, or artemether to rats during a sensitive stage of organ development, Clark 2009 [24] observed embryo lethality and birth defects, including abnormal long bones and ventricular septal defects. Similarly, artemisinin exposure during embryonic stages of the zebrafish was shown to cause severe morphological defects, like pericardial edema and curvature abnormalities [25].

Although the treatments with IC26 and IC33 altered the angle of body curvature, they did not affect the overall length of the larvae. These findings suggest that the control molecules lacking the peroxide bond may exert toxicity and teratogenic effects in zebrafish larvae through peroxide-independent mechanisms, potentially associated with their crown ether structure and metabolic pathways in the organism. Deeper structural analyses of the ethers, as well as further biological studies, may shed light on the possible mechanisms involved.

Given that morphological deformities can lead to abnormal behaviours, such as reduced locomotion, we observed that locomotor activity was suppressed not only by IC26 and IC33 but also by the 1,2,4,5-tetraoxanes YB11 and T2. These results indicate that the behavioural effects do not depend solely on the presence or absence of the peroxide bond, pointing to other mechanisms likely induced by other structural features of these compounds that influence their interaction with specific biomolecular targets of the organism, or with other factors, such as the ability to be absorbed and/or metabolized.

Several studies suggested that artemisinin and its derivatives affect SERCA activity, as evidenced by the observed dysregulation of intracellular calcium levels [6,7,34,38]. In our study, we wanted to study directly the SERCA protein expression. It is known that artemisinin and its derivates inhibit *PfATP6* [4,5] that encodes a P-type Ca^2+^-ATPase in *P. falciparum* that preserves the functional motifs of SERCA pumps and shares ~39% overall sequence identity with rabbit SERCA1a [39], whereas zebrafish *atp2a1* encodes the SERCA1 ortholog, which is strongly conserved with the human *ATP2A1* gene (NCBI Gene).

Exposure to YB1 and IC22 downregulated *atp2a1* expression, which interferes with calcium reuptake into the sarcoplasmic reticulum (Ca^2+^-ATPase), thereby affecting muscle contractility and energy homeostasis in developing larvae [40]. This disruption may contribute to early developmental toxicity and suggests a coordinated impairment in musculoskeletal differentiation. Notably, both YB1 and its ether analogue, IC22, decreased *atp2a1* expression in zebrafish larvae, whereas YB17, the tetraoxane in salt form, did not. This suggests that small structural differences between the compounds influence their effects on calcium transport and cellular uptake. It is noteworthy that *atp2a1* and *oc2* were upregulated after treatment with YB17, suggesting a compensatory response of the skeletal system. This may indicate activation of feedback mechanisms to maintain calcium balance and osteoblast development.

IC22 also affected bone marker genes for immature osteoblasts, the transcription factor *sp7*, and for mature osteoblasts *oc2*, impacting the intermediate and late osteoblast differentiation stages [41], whereas YB1 specifically reduced the expression of *oc2* in mature osteoblasts. Such downregulation of key genes for osteoblast function and differentiation indicates a potential negative impact on skeletal ossification. It is noteworthy that the transcriptional repression of IC22 was more extensive and stronger than that of YB1 at various stages of differentiation, indicating a stage-independent interference with osteoblast differentiation.

Furthermore, the transcription factor *runx2b*, which is crucial in the specification of the osteoblast, was also downregulated by YB1, and even more by IC22, reflecting an impairment of early stages of osteoblastic differentiation. This trend was also observed in the downregulation of *sp7* and *oc2* by IC22, so it appears to negatively regulate different stages of the osteoblast differentiation pathway.

Additionally, the expression of *col1a1a*, which is the major collagen component of the bone extracellular matrix, was decreased following the exposure to YB1, IC22 and YB17. This result indicates that all tested artemisinin inspired compounds caused limitation in the formation of the extracellular matrix.

The gene *alpl*, a marker of osteoblast maturation and mineralization, was downregulated by YB17, together with *col1a1a*, suggesting that YB17 cause an effect on osteoblast activity and extracellular matrix formation and affect the mineralization capacity of osteoblasts by reducing the availability of inorganic P [42].

IC22, which does not possess the peroxide bond, was found to result in the higher transcription level of repression of the osteogenic genes, in comparison to YB1, which possesses the peroxide bond. Although exposure to YB1, YB17, and IC22 did not induce evident morphological or behavioural toxicity at the tested concentrations, these alterations in gene expression raise the possibility that more pronounced variation could occur with compounds that were not tested due to less promising profiles. Investigating the gene expression alterations in zebrafish larvae after the exposure to compounds that induce morphological and behavioural toxicity could provide valuable insights into the mechanisms underlying musculoskeletal toxicity.

In many antimalarial contexts, peroxide cleavage (e.g., via ferrous iron/heme) causes ROS which damage parasites [43]. The peroxide may be metabolized by detoxification pathways (e.g., via glutathione, heme oxygenases, other reductases) before causing radical-mediated damage to the host, minimizing ROS accumulation especially if antioxidant defences are competent. Modern views of ROS emphasize that low-to-moderate ROS can act as signaling molecules, triggering adaptive responses (antioxidant gene induction, metabolic reprogramming, autophagy, survival pathways) rather than irreversible damage [44]. Although classical literature on endoperoxide antimalarials implicates peroxide-bridge cleavage and resultant radical/ROS generation as the primary cytotoxic mechanism, more recent studies in mammalian cells show that artemisinin can paradoxically act as a cytoprotective agent under oxidative challenge by reducing ROS, preventing lipid peroxidation and enhancing antioxidant enzyme activities (SOD, CAT, GPx)—likely via activation of redox-sensitive signalling pathways such as PI3K/Akt or AMPK/NRF2 [45,46].

In our experimental model, YB17 did not alter the expression of *sod1* and *cat*, suggesting that ROS generated by peroxide-bridge cleavage were likely minimal, or were efficiently reduced by endogenous defences, possibly due to the salt form of the compound and solubility/bioavailability aspects. Even without visible deformities, the treatments with tetraoxane YB1 and ether IC22 appeared to reduce the antioxidant defence system by downregulating the *cat*, an enzyme which converts hydrogen peroxide (H_2_O_2_) into water and oxygen. Alternatively, the observed upregulation of *sod1*, a gene involved in the antioxidant defence system that converts the superoxide radical (O_2_^−^) into H_2_O_2_, indicates that YB1 may have activated adaptive antioxidant signalling, leading to redox homeostasis rather than persistent oxidative stress. To gain deeper insight into possible adaptive antioxidant responses, future studies should assess antioxidant-related gene expression in the additional compounds tested, especially those that demonstrated higher toxicity compared with YB1 and YB17.

A mechanistic outcome of note is that the crown ether IC22, lacking peroxide bonds, was the most effective transcriptional repressor of musculoskeletal and oxidative stress genes. One possible explanation is that IC22, lacking the redox-active moiety, would be subject to alternative metabolisms which lead to accumulation of toxic intermediates.

The minimal activity of YB17 highlights that the salt form and altered physicochemical properties may reduce cellular uptake or modify metabolic activation, thereby minimizing its biological effect.

Despite the fact that our study points to the potential of tetraoxanes to modulate bone metabolism, a number of limitations have to be considered. First, in this study we analyzed the expression of a relatively small number of skeletal-related genes, and further research should involve screening for a wider panel of genes. Of particular interest are genes associated with the Wnt/β-catenin pathway, well recognized as central regulators of osteoblast differentiation and skeletal homeostasis. Secondly, it would be relevant to further investigate the contribution of ROS as mediators of the effects exerted by these compounds.

More assays should therefore be conducted to assess the activity of pre-osteoblasts and their differentiation in vitro in established cell systems. Such experiments would yield more mechanistic information on the action of our molecules on osteogenesis and on the function of bone cells. Future studies should also (1) involve the characterization of selective gene expression profiling and establish structure–activity connections, amplifying the chemical diversity by structural modifications of the tetraoxane-derived compounds, such as the introduction of amide or ester functional groups, or by preparing salt forms, and their biological effects; (2) elucidate the difference between metabolic pathways of endoperoxides and their corresponding ether analogues lacking the peroxide bond to gain insight into their anti-osteogenic and redox potentials; and (3) extend studies in the development of zebrafish into later stages to assess both skeletal and systemic long-term effects and to investigate potential toxicity in additional organs to determine if the observed effects are bone-specific or indicative of general toxicity.

## 5. Conclusions

In conclusion, these findings indicate that, in tetraoxanes, the peroxide groups alone do not lead to anti-osteogenic activity or systemic toxicity. However, when the tetraoxane-based molecule also incorporated an amide substituent, an anti-osteogenic effect was observed at lower concentrations. Thus, the morphological alterations and gene expression may not depend solely on the presence or absence of the peroxide bond, but rather on other features of the molecular structure impacting on absorption and metabolism, at the tested concentrations.

Also, we proved through gene expression analysis that *atp2a1*, a gene that encodes the key regulator of calcium homeostasis, *sarco*/*endoplasmic reticulum Ca^2+^-ATPase 1*, was downregulated by the most effective compounds, causing a disturbance of calcium handling and resulting in subsequent musculoskeletal effects. However, *atp2a1* does not appear to be the primary target in all cases, as exemplified by YB17, a tetraoxane in salt form. Additionally, we observed that *col1a1a*, which encodes type I collagen, was downregulated by all tested compounds. This finding suggests that *col1a1a* may represent a complementary target contributing to the anti-osteogenic potential of artemisinin-inspired compounds.

Even though morphological deformities were not visible, gene expression analysis allowed us to conclude that oxidative stress and the antioxidant defence system were affected following exposure to 1,2,4,5-tetraoxane YB1 and ether IC22. Investigating gene expression changes in zebrafish larvae after exposure to compounds that induce morphological and behavioural toxicity could provide valuable insights into the mechanisms underlying musculoskeletal toxicity. From all the assessment made here, YB17 did not alter zebrafish larval behaviour and produce minor changes in the expression of the genes analyzed like *oc2* and *col1a1a*, supporting the exhibited anti-osteogenic activity, even at nanomolar concentrations. Therefore, YB17 emerges as the most promising therapeutic candidate among the molecules tested for possible application in conditions where a controlled inhibition of bone formation is clinically beneficial, such as heterotopic ossification, fibrodysplasia ossificans progressiva [47], sclerosteosis and van Buchem disease [48]. Further studies are needed to clarify how the tested compounds act in relation to the specific underlying disease mechanisms.

All together, these findings place tetraoxane endoperoxides as lead molecules for peroxide-based therapeutic discovery. With structural optimizations achieved by adjustment of functional group substituents and the possibility of preparing salts, it should be possible to create chemical entities with skeletal selectivity and reduced systemic toxicity, as well as to obtain better mechanistic insight in the modulation of bone metabolism.

## Figures and Tables

**Table 1:** IUPAC names, internal compound codes and structures of endoperoxides (tetraoxanes) and their ether analogues.

IUPAC Name	Compound Code	Structure
**ethyl (1S,3R,5r)-dispiro[adamantane-2,2′-[1,3,4,6]tetraoxane-5′,1″-cyclohexane]-4″-carboxylate**	YB1	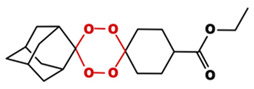
**methyl 3-methyl-2-{[(1S,3R,5r)-dispiro[adamantane-2,2′-[1,3,4,6]tetraoxane-5′,1″-cyclohexan]-4″-yl]formamido}butanoate**	YB9	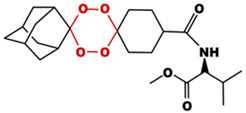
**(1S,3R,5r)-dispiro[adamantane-2,2′-[1,3,4,6]tetraoxane-5′,1″-cyclohexane]**	YB11	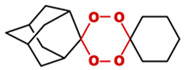
**(1S,3R,5R)-dispiro[adamantane-2,2′-[1,3,4,6]tetraoxane-5′,1″-cyclohexan]-4′-aminium 4-methylbenzenesulfonate**	YB17	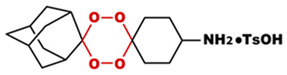
**1-{[(1S,3R,5r)-dispiro[adamantane-2,2′-[1,3,4,6]tetraoxane-5′,1″-cyclohexan]-4″-yl]carbonyl}-1H-pyrazol-3-amine**	T2	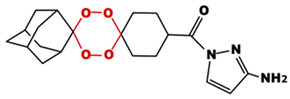
**ethyl (1S,3R,5r)-dispiro[adamantane-2,5′-[1,3]dioxane-2′,1″-cyclohexane]-4″-carboxylate**	IC22	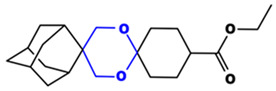
**(1S,3R,5r)-dispiro[adamantane-2,5′-[1,3]dioxane-2′,1″-cyclohexan]-4″-amine**	IC26	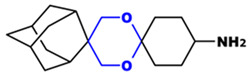
**(1S,3R,5r)-dispiro[adamantane-2,5′-[1,3]dioxane-2′,1″-cyclohexane]**	IC33	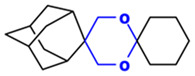

**Table 2:** LC_50_ values calculated for each tested compound. The values represent LC_50_ 72 h after exposure to each compound.

Compound	LC_50_ (µM)	Compound	LC_50_ (µM)
YB1	33.4	IC22	24.6
YB9	5.0	T2	5.2
YB11	38.3	IC33	39.4
YB17	36.5	IC26	74.4

**Table 3:** Comparative results of each treatment at the minimal concentration causing anti-osteogenic effects compared to the negative control. Statistical significances represented by asterisks; *p* < 0.05 (*), *p* < 0.01 (**), *p* < 0.0001 (****).

Compound (µM)	Osteogenic Assay	Behaviour Assay	Larval Length	Body Curvature
YB1 (6)	*	-	-	-
IC22 (20)	*	-	-	-
YB17 (0.5)	**	-	-	-
IC26 (60)	*	****	-	**
YB11 (6)	*	****	-	-
IC33 (40)	**	**	-	****
T2 (2)	*	*	-	-
YB9 (8)	**	-	-	-

## Data Availability

The original contributions presented in this study are included in the article/Supplementary Material. Further inquiries can be directed to the corresponding authors.

## Supplementary Materials

The following supporting information can be downloaded at https://www.mdpi.com/article/10.3390/toxics14030261/s1, Section S1. Synthetic procedures and experimental details for the synthesis and chemical characterization of compounds. Section S1.1. General Procedure 1: Synthesis of 1,2,4,5-Tetraoxanes YB1, YB11 and YB16. Scheme S1. Synthetic approach to 1,2,4,5-tetraoxanes (YB1, YB11, YB16, and YB17•TsOH) [49]. Section S1.2. General Procedure 2: Synthesis of the 1,2,4,5-tetraoxanes T1, T2 and YB9. Scheme S2. Synthetic approach to 1,2,4,5-tetraoxanes (T1, T2, and YB9) [50]. Section S1.3. General Procedure 3: Synthesis of the non-peroxide-containing analogues IC22, IC25 and IC33. Scheme S3. Synthetic approach to 1,2,4,5-tetraoxane controls (IC22, IC25, IC26, and IC33) [49,51]. Section S2. Detailed synthesis and characterization of the compounds [49,52,53]. Figure S1. Concentration–response curves for LC_50_ determination of tested compounds. (A) Mortality (%) of zebrafish larvae after 72 h exposed to various concentrations (µM) of compounds YB1, YB9, YB11, and YB17. (B) Mortality (%) of zebrafish larvae after 72 h exposed to various concentrations (µM) of compounds T2, IC22, IC26, and IC33. Data points represent observed mortality after 72h of the exposure and solid lines show nonlinear regression (four parameter mode) used to calculate LC_50_ values. Concentration is shown on a logarithmic scale. Figure S2. Illustration of total length measurement (A) and the angle of body curvature (B) of zebrafish larvae treated by IC33 40 µM. Scale bar: 1 mm. Figure S3. Illustration of zebrafish larvae stained with alizarin red S and illustration of measurement of normalized operculum area of 3 zebrafish larvae treated with: (A and B) YB17 0.5 µM; (C and D) dimethyl sulfoxide (DMSO); (E and F) calcitriol. The head area is outlined in yellow, and the operculum area in blue. Scale bar: 100 µm. Table S1: Sequences of primers used. All sequences in 5′–3′ orientation.

## Abbreviations

ROS	Reactive oxygen species
PfATP6	*Plasmodium falciparum* ATPase 6
ATP	Adenosine triphosphate
SERCA	Sarco/endoplasmic reticulum Ca^2+^-ATPase
PMCA4b	Plasma membrane Ca^2+^-ATPase 4b
PFA	Plakortide F acid
Pmc1	Plasma membrane calcium ATPase 1
Pmr1	P-type Ca^2+^/Mn^2+^ ATPase 1
TLC	Thin layer chromatography
NMR	Nuclear magnetic resonance
LC_50_	Lethal concentration for 50% mortality
dpf	Days post-fertilization
DMSO	Dimethyl sulfoxide
WT	Wild type
RNA	Ribonucleic acid
RNase	Ribonuclease
DNase	Deoxyribonuclease
qPCR	Quantitative polymerase chain reaction
cDNA	Complementary deoxyribonucleic acid
H_2_O_2_	Hydrogen peroxide
O_2_^−^	Superoxide radical
