# Supplementary material for: Evaluating Toxicity and Anti-Osteogenic Activity of Artemisinin-Inspired Endoperoxides in Zebrafish Larvae
Source: Toxics. 2026 Mar 17;14(3):261. doi: 10.3390/toxics14030261 (PMC13030048; doi:10.3390/toxics14030261)
Supplement: Supplementary file 1 [file toxics-14-00261-s001.zip › toxics-4140339-supplementary.pdf]

toxics

MDPI

Supplementary Material

Evaluating Toxicity and Anti-Osteogenic Activity of Artemisinin-inspired Endoperoxides in Zebrafish Larvae

Yaryna S. Buzan[1,2], Gil Martins [1], Bruno M. S. Ferreira[1,3], Inês C.C. Costa[1,2], Maria L. S. Cristiano[1,2,*], Paulo J. Gavaia[1,3,*]

1 Centro de Ciências do Mar do Algarve (CCMAR/CIMAR-LA), Campus de Gambelas, University of Algarve, 8005-139 Faro, Portugal;
2 Department of Chemistry and Pharmacy, Faculty of Sciences and Technology, FCT, Campus de Gambelas, University of Algarve, 8005-139 Faro, Portugal;
3 Faculty of Medicine and Biomedical Sciences, Campus de Gambelas, University of Algarve, 8005-139, Faro, Portugal
* Correspondence: mcristi@ualg.pt; pgavaia@ualg.pt

S1. Synthetic procedures and experimental details for the synthesis and chemical characterization of compounds.

S1.1. General Procedure 1: Synthesis of 1,2,4,5-Tetraoxanes YB1, YB11 and YB16.

Procedure adapted from Amado *et al.* [49]. Step 1 *(Peroxidation):* Carbonyl compound 1 (1 mmol) was dissolved in acetonitrile (CH_3CN, 3 mL) and formic acid (3 mL) was added. Hydrogen peroxide 50 *wt* % (H_2O_2, 4 mmol) was slowly added over an ice bath until consumption of the starting material (2 hours). To this mixture was added distilled water. The aqueous layer was rinsed with dichloromethane (DCM, 3 × 30 mL), and the combined organic extracts were washed with brine, dried over anhydrous magnesium sulfate ($MgSO_4$), filtered and concentrated under reduced pressure, at low temperature (30-35 °C), to obtain the *gem*-dihydroperoxide semi-crude, which was used immediately, without further purification. Step 2 *(Cyclocondensation):* The *gem*-dihydroperoxide semi-crude was dissolved in anhydrous DCM (DCMa) (5 mL), followed by addition of the 2-adamantanone (1.5 mmol) and bismuth(III) trifluoromethanesulfonate ($Bi(OTf)_3$, 0.05 mmol). The reaction mixture was stirred under nitrogen atmosphere (N_2) and over an ice bath to room temperature (rt) until consumption of the starting material (usually overnight [o/n]). The resulting solution was concentrated under reduced pressure and purified by flash chromatography using an ethyl acetate (EtOAc)–hexane gradient to afford pure 1,2,4,5-tetraoxanes.

Academic Editor: Firstname Lastname

Received: date
Revised: date
Accepted: date
Published: date

Copyright: © 2026 by the authors. Submitted for possible open access publication under the terms and conditions of the Creative Commons Attribution (CC BY) license.

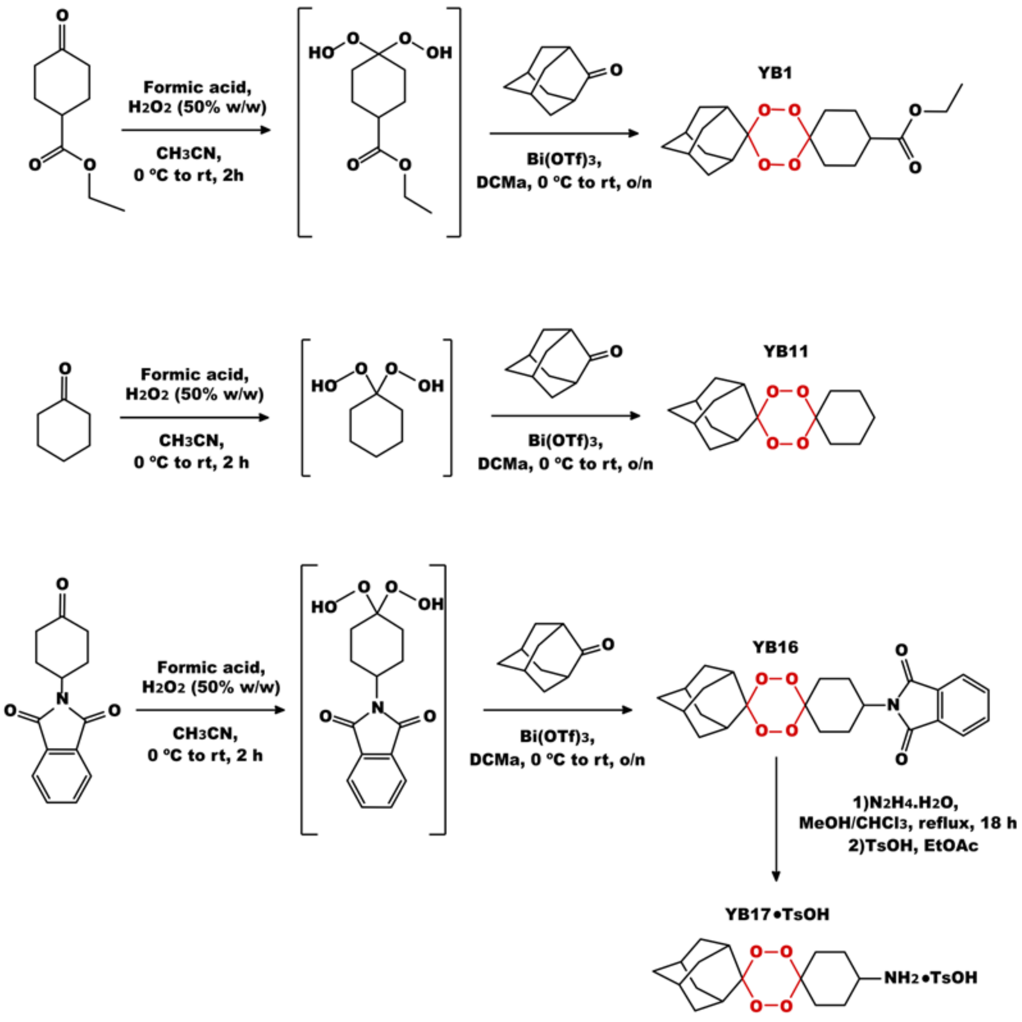

Scheme S1. Synthetic approach to 1,2,4,5-tetraoxanes (**YB1**, **YB11**, **YB16**, and **YB17•TsOH**).

S1.2. General Procedure 2: Synthesis of the 1,2,4,5-tetraoxanes T1, T2 and YB9.

Followed the procedure by Stec *et al*. [50] with slight modifications. Peroxide–carboxylic acid (1 mmol) (YB2) was dissolved in DCMa (5 mL) at room temperature. To the solution was added hydroxybenzotriazole (HOBt, 1.5 mmol), 1-ethyl-3-(3-(dimethylamino)propyl)carbodiimide hydrochloride (EDC·HCl, 1.5 mmol) and triethylamine (ET_3N, 2 mmol) under N_2. After stirring for 1 hour, the amine (1.5 mmol) was added, and the reaction mixture was stirred at room temperature until consumption of the starting material (usually overnight). To the resulting solution was added distilled water, and the mixture was extracted with DCM (3 × 15 mL). The organic layer was washed with brine, dried over anhydrous $MgSO_4$, filtered, and concentrated under reduced pressure. The residue was purified by flash chromatography using an EtOAc–hexane gradient to afford pure 1,2,4,5-tetraoxanes.

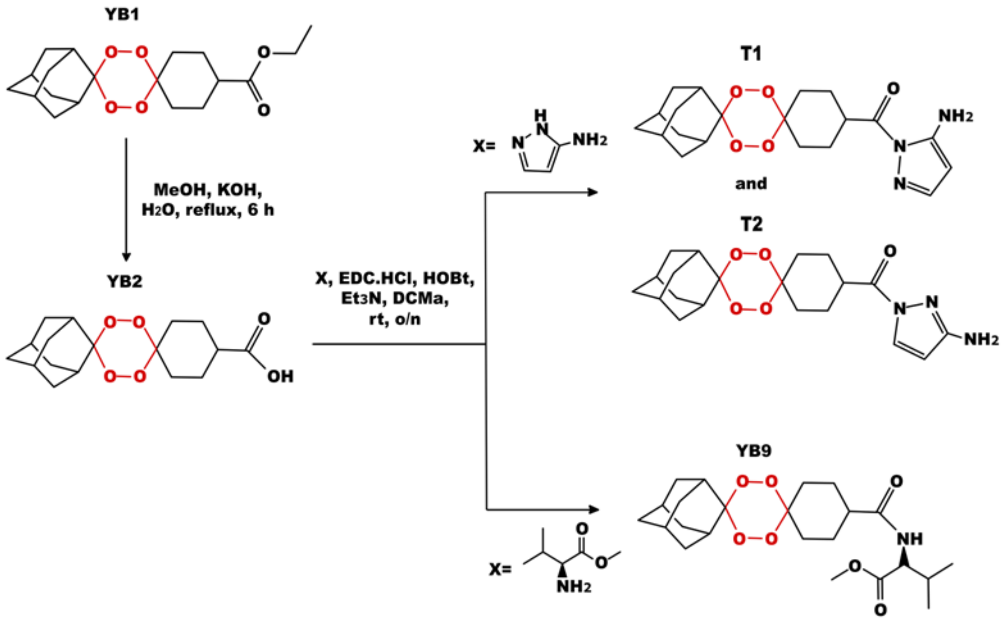

Scheme S2. Synthetic approach to 1,2,4,5-tetraoxanes (**T1**, **T2**, and **YB9**).

S1.3. General Procedure 3: Synthesis of the non-peroxide-containing analogs IC22, IC25 and IC33.

The compounds IC22, IC26, and IC33 were kindly provided by Inês C. C. Costa and have not yet been published or described in the literature.

Followed the procedure by Amado *et al* [49] and Chadwick *et al* [51]. Sodium bis(trimethylsilyl)amide (NaHMDS, 2M in tetrahydrofuran [THF]) (12 mmol) was added to a stirring solution of methoxymethyl triphenylphosphonium chloride ($MeOCH_2P^+Ph_3Cl^-$, 12 mmol) in anhydrous THF (20 ml) at 0 °C. The mixture was stirred at 0 °C for 15 minutes before a solution of 2-adamantanone (8 mmol) in anhydrous THF (5 ml) was added. The mixture was stirred at 0 °C for 2 hours before being quenched with distilled water (H_2O, 25 ml) and extracted with diethyl ether (Et_2O, 3 × 30 ml). The organic extracts were combined, dried over $MgSO_4$, filtered and concentrated under reduced pressure. The resulting residue was taken up in CH_3CN (50 ml) and 1M aq. hydrochloric acid (HCl, 12,5 ml) was added. The mixture was stirred at room temperature overnight before being concentrated under reduced pressure to remove most of the CH_3CN, partitioned between Et_2O (30 ml), followed by its saturation with aq. sodium chloride (NaCl, 20 ml). The aqueous layer was extracted with Et_2O (3 × 30 ml), and the organic extracts were combined, dried over $MgSO_4$, filtered and concentrated under reduced pressure to give a colorless oil. Purification by flash column chromatography (EtOAc−hexane, 10:90, *v*/*v*) gave (1*r*,5*R*,7*S*)-2-adamantanecarbaldehyde (91 %) as a white solid.

Subsequently, 37 % aq. formaldehyde (CH_2O, 11 mmol) was added to a stirring solution of 2-adamantanecarbaldehyde (4 mmol) in THF (1 ml) and methanol (MeOH, 1,2 ml) at 0 °C. 25 % aq. sodium hydroxide (NaOH, 5 mmol) was then carefully added and the mixture allowed to warm to room temperature and stirred overnight. The reaction mixture was poured into saturated aq. NaCl (20 ml) and extracted with THF−EtOAc (1:1, 40 ml). The organic extracts were dried over $MgSO_4$, filtered and concentrated under reduced pressure to give a white solid. Recrystallisation from Et_2O−hexane gave [(1*r*,5*R*,7*S*)-2-(Hydroxymethyl)-2-adamantanyl]methanol (98 %) as colorless needles.

The diol (1 mmol) was dissolved in DCMa (5 mL), followed by addition of the carbonyl compound (1 mmol) and silica sulfuric acid (H_2SO_4-SiO_2, 2 mmol). The reaction mixture was stirred under nitrogen atmosphere (N_2) and over an ice bath to room

temperature until consumption of the starting material. The resulting solution was concentrated under reduced pressure and purified by flash chromatography using an EtOAc–hexane gradient to afford pure compounds.

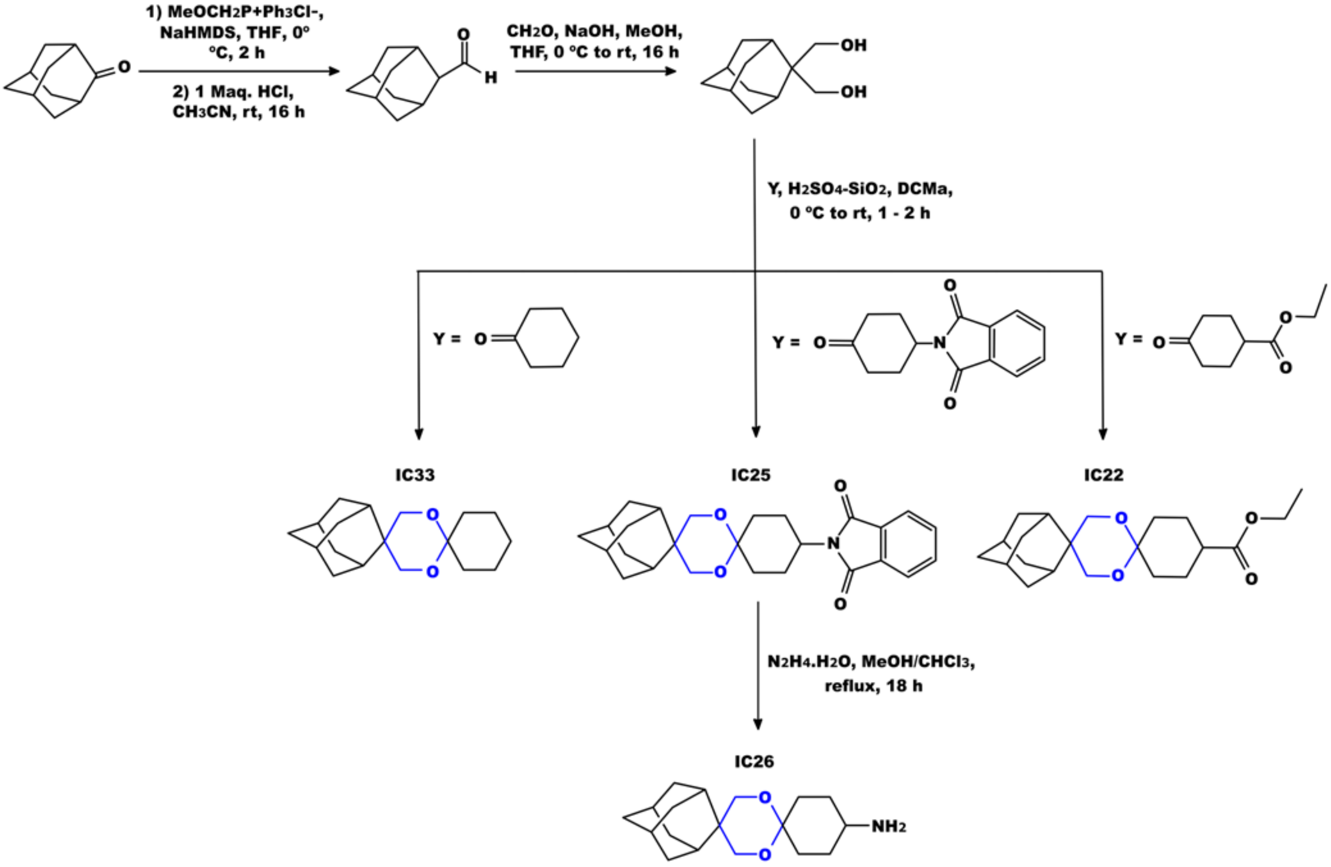

Scheme S3. Synthetic approach to 1,2,4,5-tetraoxane controls (**IC22**, **IC25**, **IC26**, and **IC33**).

S2. Detailed synthesis and characterization of the compounds

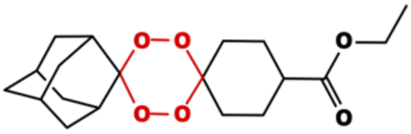

YB1 (ethyl (1S,3R,5r)-dispiro[adamantane-2,2'-[1,3,4,6]tetraoxane-5',1''-cyclohexane]-4''-carboxylate) - This compound was synthesized in accordance with general procedure 1 using 2-ethyl 4-oxocyclohexanecarboxylate (for the peroxidation step) and 2-adamantanone (for the cyclocondensation step). Purification by flash chromatography (EtOAc–hexane, 1:99, *v*/*v*) provided a white solid (46 % yield). M.p. = 74-75 °C. Spectral data are in accordance with the data reported in the literature [49]. $^{13}C\{^{1}H\}$ NMR (126 MHz, $CDCl_3$) δ 174.61, 110.48, 107.15, 60.39, 46.94, 41.64, 39.62, 38.22, 36.89, 36,26, 33.09, 29.67, 27.40, 27.00, 14.18. ^{1}H NMR (500 MHz, $CDCl_3$): δ 4.02 (s, 2H, H-x), 2.50–1.90 (m, 12H, CH_2), 1.90–1.10 (m, 10H, CH_2), 1.25 (t, 3H, CH_3). HRMS (ESI+, *m*/*z*) calcd $C_{19}H_{28}O_6Na$ $(M+Na)^+$: 375.178; found 375.177.

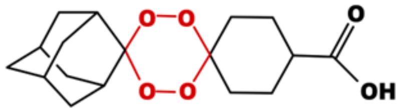

YB2 ((1S,3R,5r)-dispiro[adamantane-2,2'-[1,3,4,6]tetraoxane-5',1''-cyclohexane]-4''-carboxylic acid)—followed procedure by Cabral *et al*. [52]. To a solution of YB1 (4 mmol) in MeOH (15 mL) was added a solution of potassium hydroxide (KOH, 20 mmol) in H_2O (6 mL). The mixture was refluxed for 6 hours at 60 ºC. Then, the solution was allowed to cool to room temperature and was concentrated under reduced pressure. The crude was dissolved in water (50 ml) and then washed with DCM (30 ml). The aqueous layer was acidified to pH 1 with concentrated HCl and then extracted with DCM (3 x 40 ml). The combined organic phases were washed with brine, dried over $MgSO_4$, filtered, and concentrated under reduced pressure to give the pure compound as a white solid (95 % yield). M.p.= 179-181 °C. 13C{1H} NMR (126 MHz, $CDCl_3$) δ 175.65, 105.89, 102.37, 42.28, 36.55, 32.24, 31.61, 28.45, 22.75, 22.35. ^{1}H NMR (500 MHz, $CDCl_3$): δ 2.10–1.40 (m, 23H). HRMS (ESI+, *m*/*z*) calcd $C_{17}H_{24}O_6Cl^-$; $(M\text{-}Cl)^-$: 359.33; found 359.61

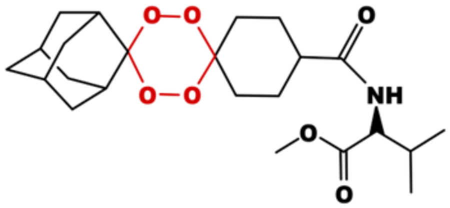

YB9 **(**methyl 3-methyl-2-{[(1S,3R,5r)-dispiro[adamantane-2,2'-[1,3,4,6]tetraoxane-5',1''-cyclohexan]-4''-yl]formamido}butanoate)—this compound was synthesized in accordance with general procedure 2 using L-Valine Methyl Ester HCl as the amine. Purification by flash chromatography (EtOAc–hexane, 20:80, *v*/*v*) provided a white solid (30% yield). M.p. = 99-101°C. ^{13}C{^{1}H} NMR (126 MHz, $CDCl_3$) δ 174.42, 172.80, 110.68, 107.20, 56.76, 52.33, 44.06, 37.05, 33.25, 32.06, 31.48, 22.83. ^{1}H NMR (500 MHz, $CDCl_3$): δ 7.25 (s, 1H), 4.57 (s, 1H), 3.70 (s, 2H), 3.33 (s, 1H), 2.01–0.75 (m, 28H). HRMS (ESI+, *m*/*z*) calcd $C_{23}H_{35}NO_7Na$ $(M+Na)^+$: 460.2302; found 460,231.

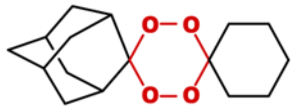

YB11 ((1S,3R,5r)-dispiro[adamantane-2,2'-[1,3,4,6]tetraoxane-5',1''-cyclohexane])—this compound was synthesized in accordance with general procedure 1 using cyclohexanone (for the peroxidation step) and 2-adamantanone (for the cyclocondensation step). Purification by flash chromatography (100% hexane) provided a yellow oil (16% yield). M.p. = 81-83 °C. Spectral data are in accordance with the data reported in the literature [49]. ^{13}C{^{1}H} NMR (126 MHz, $CDCl_3$) δ 110.43, 107.81, 37.10, 33.34, 33.28, 32.07, 29.84, 29.51, 27.20, 25.53, 22.84. ^{1}H NMR (500 MHz, $CDCl_3$): δ 3.13 (s, 1H), 2.04–1.24 (m, 23H). HRMS (MALDI-TOF, *m*/*z*) calcd for $C_{16}H_{24}O_4K$ $(M+K)^+$: 318.1311; found 318.3302.

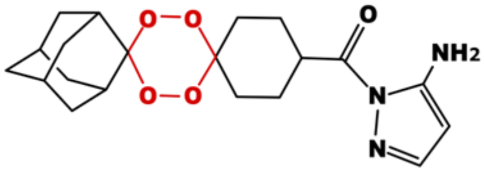

T1 (1-{[(1S,3R,5r)-dispiro[adamantane-2,2'-[1,3,4,6]tetraoxane-5',1''-cyclohexan]-4''-yl]carbonyl}-1H-pyrazol-5-amine)—this compound was synthesized in accordance with

general procedure 2 using 3-aminopyrazole as the amine. Purification by flash chromatography (EtOAc −hexane, 20:80, *v/v*) provided a white solid (17 % yield). M.p.=183-185 °C. Spectral data are in accordance with the reported in the literature [53]. $^{13}C\{^1H\}$ NMR (126 MHz, $CDCl_3$): δ 178.21, 150.40, 144.10, 110.69, 107.26, 88.99, 41.37, 37.06, 34.38, 33.27, 30.31, 29.84, 27.18, 24.92, 24.11. ^1H NMR (500 MHz, $CDCl_3$): δ 7.36 (d, J = 1.8 Hz, 1H), 5.55 (s, 2H), 5.38 (d, J = 1.8 Hz, 1H), 3.65 (ddt, J = 11.2, 7.5, 3.8 Hz, 1H), 3.17 (s, 2H), 2.21–1.59 (m, 23H). HRMS (ES+, m/z) calcd $C_{20}H_{28}N_3O_5$ $(M+H)^+$: 390.20235; found 390.20181.

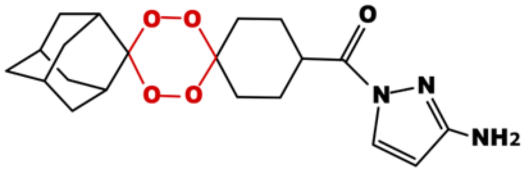

T2 (1-{[(1S,3R,5r)-dispiro[adamantane-2,2'-[1,3,4,6]tetraoxane-5',1''-cyclohexan]-4''-yl]carbonyl}-1H-pyrazol-3-amine)—this compound was synthesized in accordance with general procedure 2 using 3-aminopyrazole as the amine. Purification by flash chromatography (EtOAc −hexane, 20:80, *v/v*) provided a white solid (34 % yield). M.p.= 157-158 °C. Spectral data are in accordance with the data reported in the literature [53]. $^{13}C\{^1H\}$ NMR (126 MHz, $CDCl_3$) δ 172.83, 157.45, 130.20, 107.29, 101.09, 40.09, 37.05, 33.26, 32.05, 29.49, 27.16. ^1H NMR (500 MHz, CDCl3): δ 8.00 (br s, 1H, NH), 6.05 (s, 1H), 3.50 (m, 1H, O–CH), 2.17–1.62 (m, 25H, CH_2 and CH_3). HRMS (ESI+, *m/z*) calcd $C_{20}H_{28}N_3O_5H$ $(M+H)^+$: 390.20235; found 390.20206.

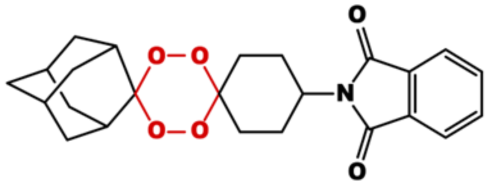

YB16 (2-[(1S,3R,5r)-dispiro[adamantane-2,2'-[1,3,4,6]tetraoxane-5',1''-cyclohexan]-4''-yl]-2,3-dihydro-1H-isoindole-1,3-dione)—this compound was synthesized in accordance with general procedure 1 using 2-(4-oxocyclohexil)isoindoline-1-3-dione (for the peroxidation step) and 2-adamantanone (for the cyclocondensation step). Purification by flash chromatography (EtOAc −hexane, 5:95, *v/v*) provided a white solid (37% yield). M.p. = 172-174 °C. Spectral data are in accordance with the data reported in the literature [49]. $^{13}C\{^1H\}$ NMR (126 MHz, $CDCl_3$) δ 168.26, 134.04, 132.06, 123.31, 110.69, 106.72, 49.79, 37.07, 33.25, 32.05, 29.79, 29.79, 27.17. ^1H NMR (500 MHz, $CDCl_3$-d): 8.00–7.35 (m, 4H, Ar–H), 4.50–4.00 (m, 1H, CH), 2.30–1.85 (m, 7H, CH_2), 1.80–1.00 (m, 12H, CH_2, CH_3), 1.00–0.80 (t, 1H, CH_3). HRMS (ESI+, *m/z*) calcd $C_{24}H_{27}NO_6$ $(M+Na)^+$: 448.1731; found 448.17.

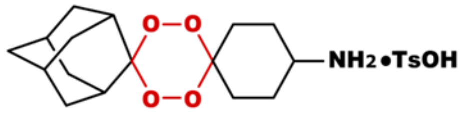

YB17•TsOH (1S,3R,5R)-dispiro[adamantane-2,2′-[1,3,4,6]tetraoxane-5′,1″-cyclohexan]-4′-aminium 4-methylbenzenesulfonate—to a solution of YB16 (2 mmol) were added hydrazine hydrate (50% *w/w*, 24 mmol) and a chloroform ($CHCl_3$)/MeOH mixture (7:3, 27 mL). The reaction mixture was refluxed at 62 °C overnight. The resulting suspension was filtered under reduced pressure to remove solid by-products, and the resultant filtrate was washed three times with hexane. To the filtrate, *p*-toluenesulfonic acid monohydrate (TsOH, 2 mmol) and EtOAc (5 mL) were added to promote salt formation. After 15 minutes, the product was isolated by filtration with cold hexane and obtained a yellow

solid was obtained. (60 % yield). M.p. = 126-128 °C. $^{13}C\{^1H\}$ NMR (126 MHz, DMSO-D_6) δ 109.87, 106.91, 47.92, 36.22, 32.58, 26.43, 20.84, 20.55. ^1H NMR (500 MHz, DMSO-D_6): δ 3.02 – 2.70 (m, 3 H, CH_2), 2.00 – 1.45 (m, 19 H, CH_2), 1.33 (m, 3 H, CH_3). HRMS (ESI+, *m*/*z*) calcd $C_{16}H_{25}NO_4H$ $(M+H)^+$: 296,1850; found 296,186.

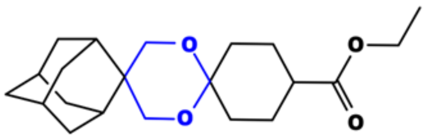

IC22 (ethyl (1S,3R,5r)-dispiro[adamantane-2,5'-[1,3]dioxane-2',1''-cyclohexane]-4''-carboxylate)—this compound was synthesized in accordance with general procedure 3 using 2-ethyl 4-oxocyclohexanecarboxylate as the carbonyl compound. Purification by flash chromatography (EtOAc: hexane, 10:90, *v*/*v*) provided a white solid. M.p. = 73-75 °C. $^{13}C\{^1H\}$ NMR (126 MHz, $CDCl_3$) δ 175.78, 97.42, 66.78, 60.62, 42.57, 39.38, 39.25, 37.94, 32.78, 32.75, 28.38, 25.45, 14.62. ^1H NMR (500 MHz, $CDCl_3$): δ 4.20–3.70 (m, 3H), 2.50–1.00 (m, 29H). HRMS (ESI+, *m*/*z*) calcd $C_{21}H_{32}O_4Na$ $(M+Na)^+$: 371.47; found 371.219.

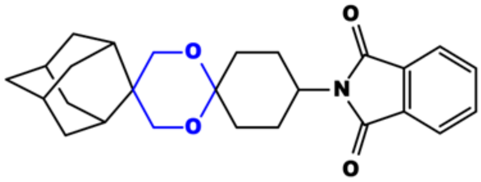

IC25 (2-[(1S,3R,5r)-dispiro[adamantane-2,5'-[1,3]dioxane-2',1''-cyclohexan]-4''-yl]-2,3-dihydro-1H-isoindole-1,3-dione)—this compound was synthesized in accordance with general procedure 3 using 2-(4-oxocyclohexil)isoindoline-1-3-dione as the carbonyl compound. The resulting solution was then filtered, extracted with DCM (3 × 50 mL), dried over with $MgSO_4$, and concentrated under reduced pressure, yielded IC25 (84 %) as a white solid. M.p., 175–177 °C. ^1H $^{13}C\{^1H\}$ NMR (126 MHz, $CDCl_3$): δ 168.4, 133.9, 132.2, 123.2, 96.7, 66.9, 66.5, 50.3, 39.0, 37.7, 32.6, 32.5, 31.8, 31.0, 28.1, 25.9. NMR (500 MHz, $CDCl_3$): δ 7.81 (dd, *J* = 5.4, 3.0 Hz, 2H), 7.69 (dd, *J* = 5.4, 3.0 Hz, 2H), 4.17 (tt, *J* = 12.4, 3.9 Hz, 1H), 3.89 (s, 4H), 2.54 – 2.45 (m, 2H), 2.38 – 2.33 (m, 2H), 1.98 – 1.85 (m, 8H), 1.70 – 1.56 (m, 8H), 1.46 – 1.39 (m, 2H).

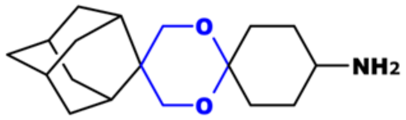

IC26 ((1S,3R,5r)-dispiro[adamantane-2,5'-[1,3]dioxane-2',1''-cyclohexan]-4''-amine)—to a solution of IC25 (2 mmol) were added hydrazine hydrate ($N_2H_4.H_2O$, 50% *w*/*w*, 24 mmol) and a $CHCl_3$/MeOH mixture (7:3, 27 mL). The reaction mixture was refluxed at 62 °C overnight. The resulting suspension was filtered under reduced pressure to remove solid by-products, and the resultant filtrate was washed three times with hexane. M.p. = 136-138 °C. $^{13}C\{^1H\}$ NMR (126 MHz, $CDCl_3$) δ 97.44, 66.69, 49.89, 39.52, 38.99, 38.64, 37.64, 32.68, 32.50, 30.98, 30.73, 28.13. ^1H NMR (500 MHz, $CDCl_3$): δ 4.00–3.70 (m, 4H), 2.80–1.00 (m, 24H). HRMS (ESI+, *m*/*z*) calcd $C_{18}H_{29}NO_2H$ $(M+H)^+$: 292.43; found 292.227.

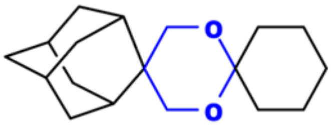

IC33 ((1S,3R,5r)-dispiro[adamantane-2,5'-[1,3]dioxane-2',1''-cyclohexane])—this compound was synthesized in accordance with general procedure 3 using cyclohexanone as the carbonyl compound. The resulting solution was then filtered, extracted with DCM (3 × 50 mL), dried over with $MgSO_4$, and concentrated under reduced pressure, giving the crude. Purification by flash chromatography (EtOAc: hexane, 1:99, *v*/*v*) provided a white solid (1.0 g, 89 %). M.p. = 95-97 °C. $^{13}C\{^1H\}$ NMR (126 MHz, $CDCl_3$) δ 98.24, 66.51, 39.28, 37.94, 32.80, 31.30, 28.41, 26.13, 23.14. ^1H NMR (500 MHz, $CDCl_3$): δ 4.05 (m, 2H), 2.10–1.00 (m, 26H). HRMS (ESI+, *m*/*z*) calcd $C_{18}H_{28}O_2Na$ $(M+Na)^+$: 299.40; found 299.198.

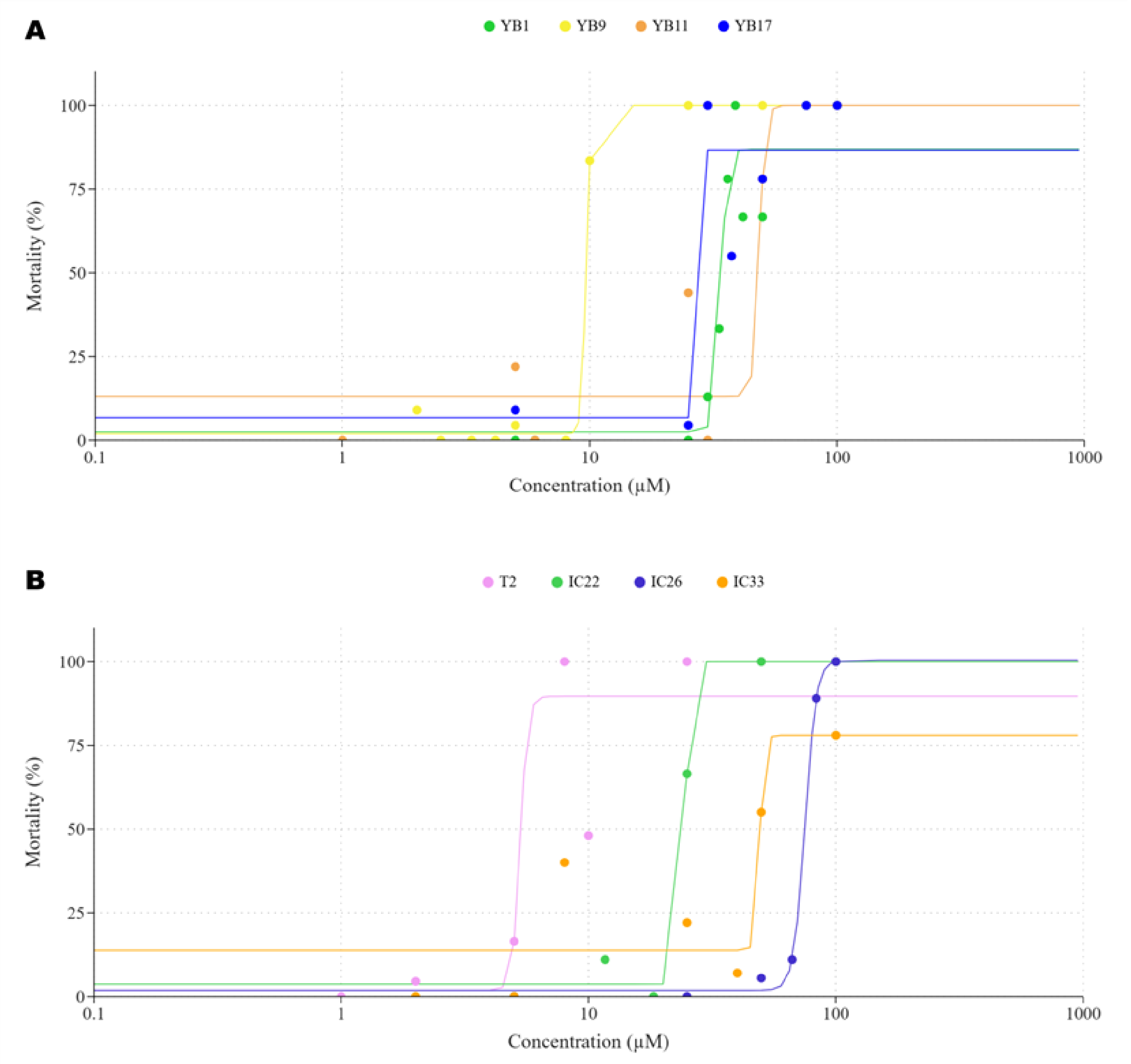

Figure S1. Concentration–response curves for LC_{50} determination of tested compounds. (A) Mortality (%) of zebrafish larvae after 72 hours exposed to various concentrations (μM) of compounds YB1, YB9, YB11, and YB17. (B) Mortality (%) of zebrafish larvae after 72 hours exposed to various concentrations (μM) of compounds T2, IC22, IC26, and IC33. Data points represent observed mortality after 72h of the exposure and solid lines show nonlinear regression (four parameter mode) used to calculate LC_{50} values. Concentrations are shown on a logarithmic scale

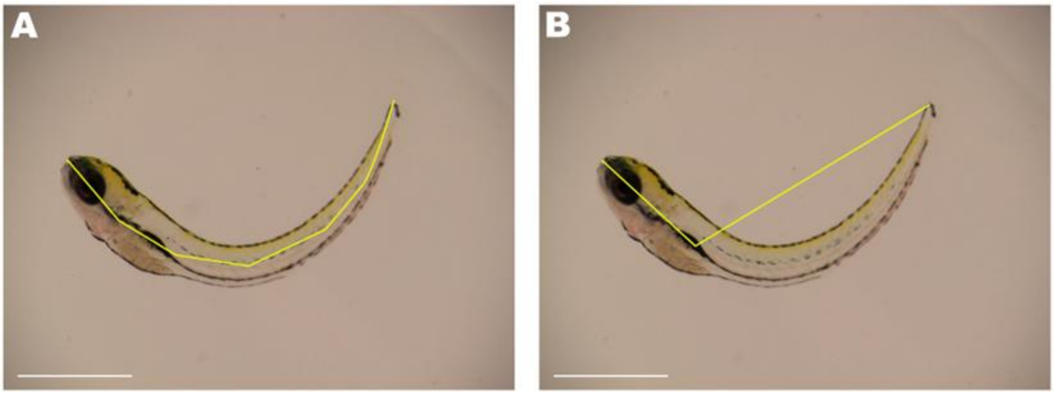

Figure S2. Illustration of total length measurement (A) and the angle of body curvature (B) of zebrafish larvae treated by IC33 at 40 µM. Scale bar: 1 mm.

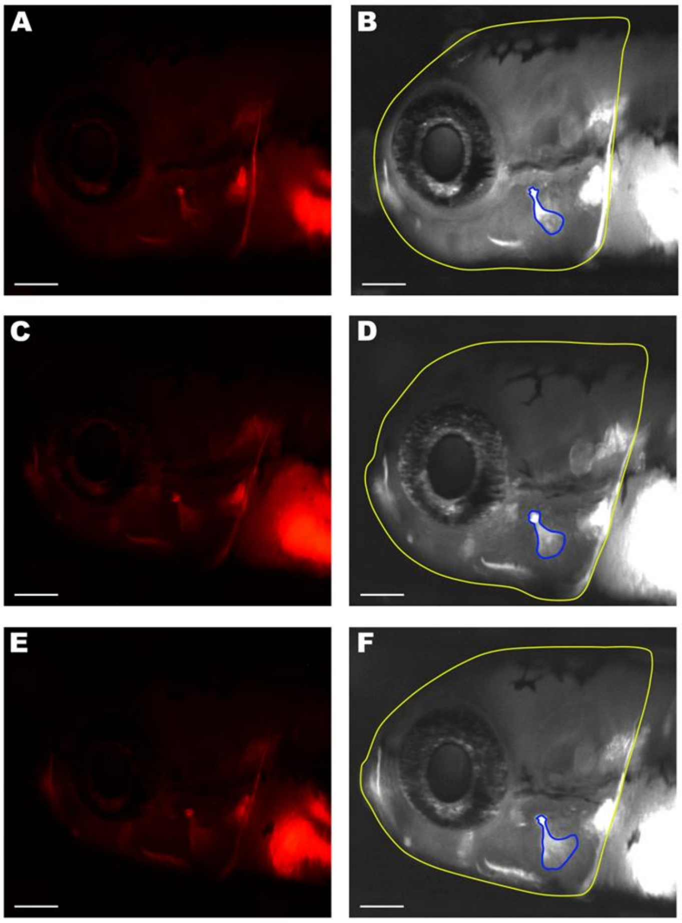

Figure S3. Illustration of zebrafish larvae stained with alizarin red S and illustration of measurement of normalized operculum area of 3 zebrafish larvae treated with: (A and B) YB17 0.5 µM; (C and D) dimethyl sulfoxide (DMSO); (E and F) calcitriol. The head area is outlined in yellow and the operculum area in blue. Scale bar: 100 µm.

Table S1. Sequences of primers used. All sequences in 5′–3′ orientation.

Gene	Primer Sequence	GenBank (Accession No.)
18s	Fw: AACACGAACATTGATGGAAGACG	NM_001423550.2
	Rev: ATTAGCAAGGACCTGGCTGTATTT	
atp2a1	Fw: GTCTGTCTACTGCTCCCCTG	NM_001007029.2
	Rev: CAGAGGGTTGTCACGGGTTG	
cat	Fw: GACTCTTCTCCTACCCGGACACA	NM_130912.3
	Rev: TAGTTTGGAGCACCACCTTGGTT	
sp7	Fw: TGGATAACTCAATGGGGCTCAA	NM_212863.2
	Rev: AGCCTTTCCAGCTCCTGACAAT	
sod1	Fw: TCCTTCTCATGAATCACCATGGTCC	NM_131294.1
	Rev: GCCAACCGATAGTGTGAGACACG	
oc2	Fw: CACTCCTGCTCCTCATGTGC	NM_001291889.2
	Rev: GTGTAAGCCGCTACGATCCC	
β-actin	Fw: TGATGCCCCTCGTGCTGTTTTC	NM_131031.2
	Rev: CTCATTGTAGAAGGTGTGATG	
ef1-α	Fw: TTGAGAAGAAAATCGGTGGTGCTG	NM_131263.1
	Rev: GGAACGGTGTGATTGAGGGAAATTC	
alpl	Fw: GATGTGGGAATGTGGATGCTTT	NM_201007.3
	Rev: CCGCTTCTCTTGCTCTGGAAA	
col1a1a	Fw: CCAGGCATCAAGGGACACAGAG	NM_199214.1
	Rev: CACCATCATTACCACGAGCACC	
runx2b	Fw: TCAGGAATGCCTCAGGGGTTATG	NM_212862
	Rev: CTTGCGGTGGGTTTGTGAATACT	
